# Non-diffraction propagation of acoustic waves in a rapidly modulated stratified medium

**DOI:** 10.1038/s41598-017-08750-z

**Published:** 2017-08-15

**Authors:** Xing-Feng Zhu, Qi Wei, Ying Cheng, Da-Jian Wu, Xiao-Jun Liu

**Affiliations:** 1 Key Laboratory of Modern Acoustics, Department of Physics and Collaborative Innovation Center of Advanced Microstructures, Nanjing University, Nanjing, 210093 China; 2 Jiangsu Key Lab on Opto-Electronic Technology, School of Physics and Technology, Nanjing Normal University, Nanjing, 210023 China

## Abstract

A rapidly modulated stratified medium with a large mass density modulation depth (LMMD) is proposed to achieve non-diffraction propagation (NDP) of acoustic waves. It is found that the NDP in LMMD medium is independent of the incident angle and can be operated in a broad-band manner. Such an NDP is robust and is unhampered by medium losses. An effective medium theory (EMT) is developed for acoustic waves propagating in the LMMD medium based on the first-principles method. The LMMD EMT is verified by using the transfer-matrix method (TMM) for both propagating and evanescent waves. Furthermore, we discuss the influence of the geometry on NDP, and finite element simulations are conducted to verify the NDP in the LMMD medium.

## Introduction

Non-diffraction propagation (NDP) of acoustic waves in spatially modulated media has been widely studied theoretically and experimentally because of the potential applications in wave beaming, acoustic waveguides, and subwavelength imaging^1–14^. The NDP of the wave beam shows flat equifrequency contours (EFCs) in the band structure; hence, the efficient NDP with a small angular divergence requires long flat EFCs^15–21^. In general, NDP appears only in a small range of incident angles on the flat segments of the EFCs and is very sensitive to the frequency of the incident waves; these features hinder the further actual applications of NDP. Therefore, many studies have been carried out to expand both the incident angle and the working frequency for NDP^10–14^. For example, in phononic crystals, the filling ratios or the form of the scatterer could broaden the frequency region of NDP^10, 11^. A rectangular phononic crystal is more favourable for expanding the incident angle range compared to a triangular one^13, 14^.

Recently, Rizza and Ciattoni^22, 23^ proposed a novel and robust regime for NDP when the transverse magnetic (TM) waves propagate in rapidly modulated stratified media with a large dielectric modulation depth (Kapitza medium)^24^. The rapid and large modulation of permittivity strongly suppresses the longitudinal component of the electric field, thus slowing down diffraction of the TM waves. Such a design is capable of achieving all-angle self-collimation, including the evanescent waves at any frequency, resulting in NDP. It is well known that the acoustic equations in a fluid are identical in form to the single polarization Maxwell equations via a variable exchange that also preserves the boundary conditions in a two-dimensional geometry. In many cases, the two acoustic parameters (mass density and bulk modulus) could be analogous to the two parameters (electric permittivity and magnetic permeability) in the electromagnetic problem^25^. Extending the concept from optics, the NDP of acoustic waves may occur in a rapidly modulated stratified medium.

In this paper, we propose a rapidly modulated stratified medium with a large mass density modulation depth (LMMD) that is capable of achieving broad-band NDP for all incident angles. An effective medium model for the LMMD medium is deduced based on the first-principles method because the very deep and rapidly modulated mass density variation entails a medium homogenization that cannot be described by the standard effective medium theory (EMT). The EMT for the LMMD medium is verified by using the transfer-matrix method (TMM) for both propagating and evanescent waves. It is found that the large mass density modulation leads to non-diffraction propagation of acoustic waves that is robust and cannot be hampered by medium losses. Furthermore, we discuss the influence of the geometry on NDP, and finite element simulations are conducted to verify NDP in the LMMD medium.

## Results

### Effective medium theory based on the first-principles method

An anisotropic acoustic metamaterial often possesses an anisotropic mass density tensor ***ρ*** and a scalar bulk modulus *κ* ^26^. Here, we only consider an inviscid fluid-like metamaterial with zero shear modulus. The anisotropic mass density tensor ***ρ*** should be expressed as diagonal $\,{\boldsymbol{\rho }}=diag[{\rho }_{x},{\rho }_{y},{\rho }_{z}]$. The propagation of acoustic waves in this material is governed by the conservation of momentum and mass equations of a general form^26^,

1 $$\{\begin{array}{c}\nabla p=-{\boldsymbol{\rho }}\frac{\partial v}{\partial t}\\ \nabla \cdot {\boldsymbol{v}}=-\frac{1}{\kappa }\frac{\partial p}{\partial t}\end{array}\,,$$

where *p* is the hydrostatic pressure field, and ***v*** is the velocity field. Inside the metamaterial, the two-dimensional pressure field *p* and velocity field ${\boldsymbol{v}}=({v}_{x},{v}_{z})$ are related by the motion equations, where time dependence *e* ^−*iwt*^ has been assumed:

2 $$\{\begin{array}{c}{\partial }_{x}p=i\omega {\rho }_{x}{v}_{x}\\ {\partial }_{z}p=i\omega {\rho }_{z}{v}_{z}\\ {\partial }_{x}{v}_{x}+{\partial }_{z}{v}_{z}=i\omega p/\kappa \end{array}.$$

Here, we consider a specific medium periodically modulated along the *z*-axis whose relative mass density ${\rho }_{r}={\rho }_{x}/{\rho }_{0}={\rho }_{z}/{\rho }_{0}$ *(ρ*_0_ is the mass density of the background medium) permits the Fourier series expansion

3 $${\rho }_{r}={\rho }_{m}+\sum _{n\ne 0}({a}_{n}+\frac{{b}_{n}}{\eta }){e}^{in(\frac{K}{\eta })z},$$

where *ρ*_*m*_ is the average of the relative mass density *ρ*_*r*_, (${a}_{n}+{b}_{n}/\eta$) is the Fourier coefficient, $2\pi \eta /K$ is the spatial period, and *η* is a dimensionless parameter. The propagation of acoustic waves is now characterized by two very different scales, i.e., a macroscopic one (the radiation wavelength) and a microscopic one (the mass density modulation period). To describe this problem, we will rely on a two-scale expansion of the fields^27, 28^. The physical problem is described by two variables: a slow coordinate *z* (macroscopic) and a fast coordinate $Z=z/\eta$ (microscopic) representing the rapid variations of the material at the scale of the basic cell, measured by *η*. It is natural to allow each acoustic field component to separately depend on the slow coordinate *z* and fast coordinate *Z*, allowing for decomposition of each component as a Taylor expansion up to first order in *η*,

4 $$H(x,z,Z)=[{\bar{H}}^{(0)}(x,z)+{\tilde{H}}^{(0)}(x,z,Z)]+\eta [{\bar{H}}^{(1)}(x,z)+{\tilde{H}}^{(1)}(x,z,Z)],$$

where *H* = *p*, *v*_*x*_, or *v*_*z*_, and the superscript (0) or (1) indicates the order of each term. The overline and tilde label the averaged and rapidly varying contributions to each order, respectively. After substituting Eq. (4) into Eq. (2) and noting that ${\partial }_{z}={\partial }_{z}+\frac{1}{\eta }{\partial }_{Z}$, each equation yields a power series in *η* whose various orders are the superposition of slowly varying (independent of *Z*) and fast (dependent on *Z*) contributions. For each order, the averaged and rapidly varying contributions can be independently balanced. From the lowest order ${\eta }^{-1}$, ${{\bar{v}}_{x}}^{(0)}=0$, ${{\tilde{v}}_{x}}^{(0)}=0$, ${{\tilde{v}}_{z}}^{(0)}=0$, and ${\tilde{p}}^{(0)}=\frac{\omega {\rho }_{0}}{K}\sum _{n\ne 0}\frac{{b}_{n}}{n}{e}^{inKZ}{{\bar{v}}_{z}}^{(0)}$ can be obtained. From the order ${\eta }^{0}$ of the third line of Eq. (2), we obtain

5 $${\partial }_{z}{{\bar{v}}_{z}}^{(0)}=i\omega \frac{1}{\kappa }{\bar{p}}^{(0)},$$

6 $${\partial }_{Z}{{\tilde{v}}_{z}}^{(1)}=i\omega \frac{1}{\kappa }{\tilde{p}}^{(0)},$$

7 $${{\tilde{v}}_{z}}^{(1)}=\frac{{k}_{0}^{2}}{{K}^{2}}\sum _{n\ne 0}\frac{{b}_{n}}{{n}^{2}}{e}^{inKZ}{{\bar{v}}_{z}}^{(0)}.$$

From the slowly varying part of the order ${\eta }^{0}$ for the second line of Eq. (2) and Eq. (7), we obtain

8 $${\partial }_{z}{\bar{p}}^{(0)}=i\omega {\rho }_{0}{\rho }_{r}^{eff}{{\bar{v}}_{z}}^{(0)},$$

where

9 $${\rho }_{r}^{eff}={\rho }_{m}+\frac{{k}_{0}^{2}}{{K}^{2}}\sum _{n\ne 0}\frac{{b}_{-n}{b}_{n}}{{n}^{2}}.\,$$

Therefore, the pressure field and velocity field in a rapidly modulated stratified medium only have slowly varying terms, i.e., $p\approx {\bar{p}}^{(0)}(x,z)$ and ${\boldsymbol{v}}\approx {{\bar{v}}_{z}}^{(0)}{\hat{e}}_{z}$. The two-dimensional motion equations of Eq. (2) become one-dimensional motion equations of Eqs (5) and (8); then, the acoustic waves propagating in the LMMD medium can be simulated by these in an effective homogeneous medium with uniform effective mass density ${\rho }_{0}{\rho }_{r}^{eff}$. It is obvious that, regardless of the medium losses, the effective mass density is not even defined and the velocity field component ${v}_{x}$ vanishes because the density is not assumed to be real. Therefore, the NDP within the LMMD medium is very robust and cannot be hampered, even in the presence of medium losses. The effective mass density of Eq. (9) has two contributions: the former corresponds to the average mass density, and the latter corresponds to the rapidly varying part of the density modulation. The proposed EMT is fundamentally different from the standard EMT^29^, whose effective mass densities are ${\rho }_{x}\,=\,< {\rho }^{-1}{ > }^{-1}$ and ${\rho }_{z}\,=\, < \rho  >$. When ${b}_{n}=0$, i.e., without the large modulation depth contribution to the density, Eq. (3) for the LMMD medium is the same as that for the standard EMT.

### Numerical demonstrations

**Figure 1:**
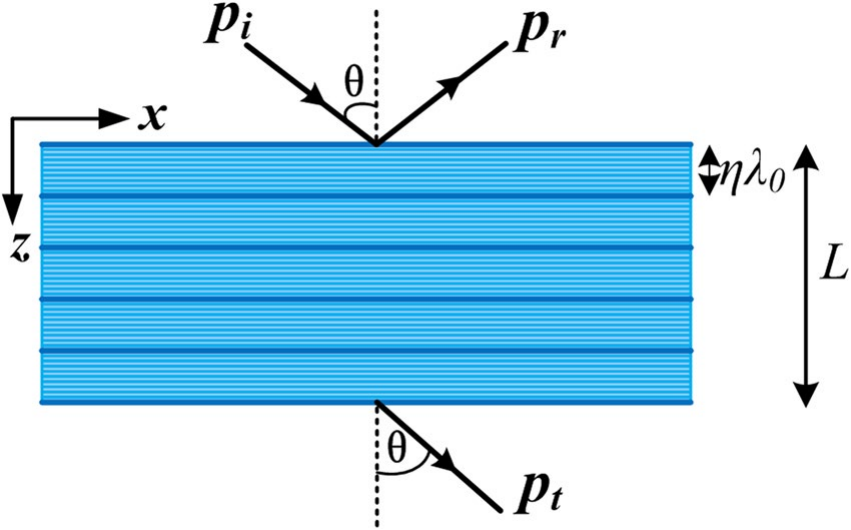
Stratified LMMD medium slab and scattering geometry of the acoustic waves.

**Figure 2:**
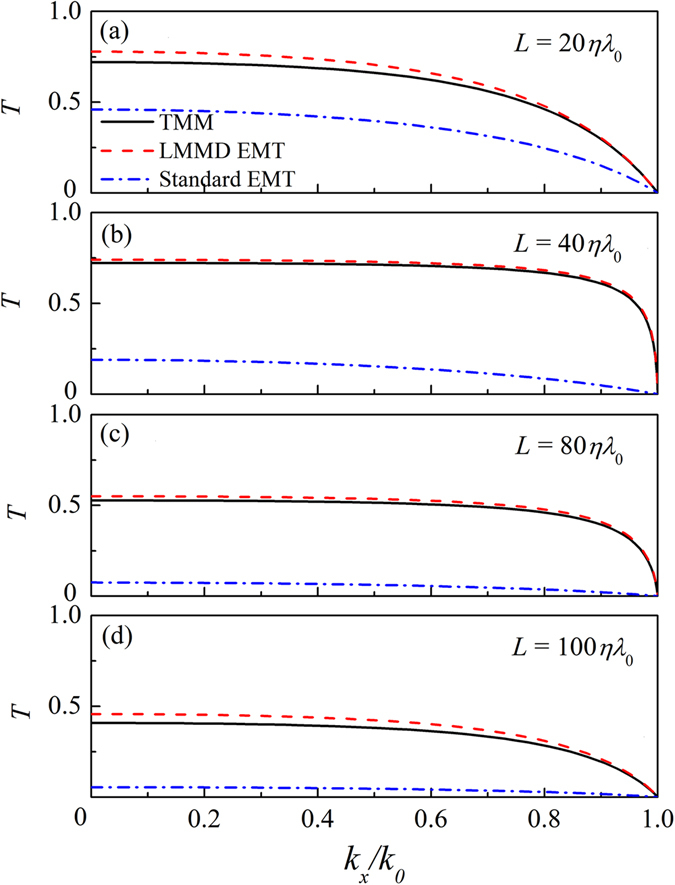
Transmissivity of the LMMD medium slab with thicknesses of (**a**) $20\eta {\lambda }_{0}$, (**b**) $40\eta {\lambda }_{0}$, (**c**) $80\eta {\lambda }_{0}$, and (**d**) $100\eta {\lambda }_{0}$ as a function of (${k}_{x}/{k}_{0}$).

To check the prediction of the proposed EMT, let us consider the reflection and transmission of acoustic plane waves from a slab filled with the LMMD medium. Figure 1 shows that the acoustic waves with an incident angle *θ* propagate from air into the layered LMMD medium slab. The mass density modulation is along the *z*-axis with a period of $\Lambda =\eta {\lambda }_{0}$. Here, ${\lambda }_{0}=2\pi /{k}_{0}$ is the incident wavelength, and *η* is the small parameter. The slab thickness *L* is a multiple of the period $\eta {\lambda }_{0}$, and the unit cell consists of *N* homogeneous layers with a thickness of $\eta {\lambda }_{0}/N$. The structural parameters of the layered LMMD medium can be designed according to the incident wavelength, which means that we can achieve the NDP at any desired frequency. Based on Eq. (3), the mass density of the *j*th layer in the unit cell ($j=1,\ldots ,N$) can be expressed as

10 $${\rho }_{r}={\rho }_{m}+(\frac{1}{\eta }+i\delta \rho )\cos \,[\frac{2\pi }{N}(j-1)],$$

where *ρ*_*m*_ is the mean value of the mass density, and $\delta \rho$ (not large) is responsible for the modulation of medium absorption. Figure 2 shows the transmissivities ($T={|{p}_{t}|}^{2}/{|{p}_{i}|}^{2}$) of the acoustic plane waves through the layered LMMD medium slabs with various thicknesses *L* as a function of ${k}_{x}/{k}_{0}=sin\theta$. The acoustic waves with an incident angle *θ* propagate from air into the layered slab. The material parameters ${\rho }_{m}=0.05+0.05i$, $\delta \rho =0.025$, $N=10$, and $\eta =1/60$. Figure 2(a),(b),(c), and (d) represent the results for the slab with various thicknesses of $20\eta {\lambda }_{0}$, 40$\eta {\lambda }_{0}$, $80\eta {\lambda }_{0}$, and $100\eta {\lambda }_{0}$, respectively. The solid lines show the transmissivities calculated by using the transfer-matrix method (TMM), which are the exact results. The dashed lines represent the results based on the proposed effective medium theory for LMMD (LMMD EMT). Based on Eq. (5) and Eq. (8), the transmissivity can be expressed as

11 $$T={|\cos ({k}_{z}L)-iFsin({k}_{z}L)|}^{-2},$$

where ${k}_{z}={k}_{0}\sqrt{{\rho }_{r}^{eff}}$ and $F=[\sqrt{{\rho }_{r}^{eff}}cos\theta +\frac{1}{\sqrt{{\rho }_{r}^{eff}}cos\theta }]/2$. Inserting the Fourier coefficients of the considered density profile into Eq. (9), we can obtain ${\rho }_{r}^{eff}$. The dash-dotted lines show the profiles of the transmissivity evaluated by using the standard effective medium theory (standard EMT). It is obvious that the results based on the LMMD EMT match well with the exact results, whereas the standard EMT is not suitable for the LMMD medium. Even within the longwave approximation regime, the standard EMT is inadequate for the layered LMMD medium because the contributions arising from the rapidly varying periodic density oscillations become important in the LMMD medium.

**Figure 3:**
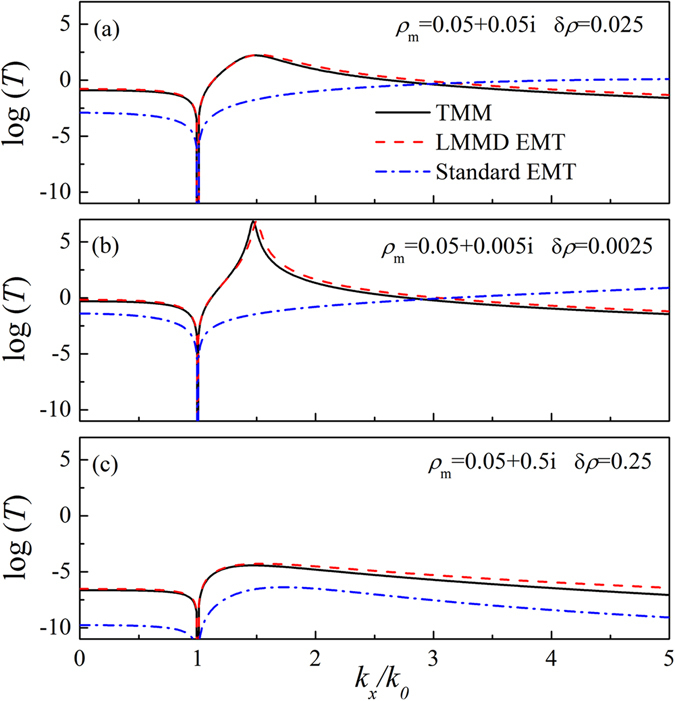
Logarithmic plot of the quantity $T={|{p}_{t}|}_{z=L}^{2}/{|{p}_{i}|}_{z=0}^{2}$ of the LMMD medium slab with (**a**) ${\rho }_{m}=0.05+0.05i$ & $\delta \rho =0.025$, (**b**) ${\rho }_{m}=0.05+0.005i$ & *δρ* = 0.0025, and (**c**) ${\rho }_{m}=0.05+0.5i$ & *δρ* = 0.25 as a function of (${k}_{x}/{k}_{0}$).

We further investigate the validity of the LMMD EMT for ${k}_{x} > {k}_{0}$. In this condition, the scattering of acoustic waves should encompass evanescent waves. The quantity $T={|{p}_{t}|}_{z=L}^{2}/{|{p}_{i}|}_{z=0}^{2}$ represents the above discussed slab transmissivity for $|{k}_{x}| < {k}_{0}$ and the efficiency in transporting evanescent waves for $|{k}_{x}| > {k}_{0}$. Figure 3(a) shows the logarithmic plot of *T* as a function of the (${k}_{x}/{k}_{0}$) value. The material parameters are identical to those considered in Fig. 2. The thickness of the slab is fixed at $100\eta {\lambda }_{0}$. The solid, dashed, and dash-dotted lines show the results obtained by using the TMM, LMMD EMT, and standard EMT, respectively. It is obvious that the LMMD EMT predictions agree well with the exact ones for both propagating and evanescent waves, whereas the standard EMT is inadequate for the LMMD medium. In addition, it is noted that medium absorption plays a very detrimental role in the NDP^30^. Figures 3(b) and (c) show the logarithmic plot of *T* for the LMMD medium slab for various absorptions with ${\rho }_{m}=0.05+0.005i$ and $\delta \rho =0.0025$ and with ${\rho }_{m}=0.05+0.5i$ and $\delta \rho =0.25$, respectively. The other material parameters are the same as those of Fig. 3(a). In these two situations, the results obtained by using the LMMD EMT approach match well with the exact ones, whereas the ones based on the standard EMT fail. Therefore, the proposed LMMD EMT is very robust against the medium losses, and this feature is very important in the NDP.

**Figure 4:**
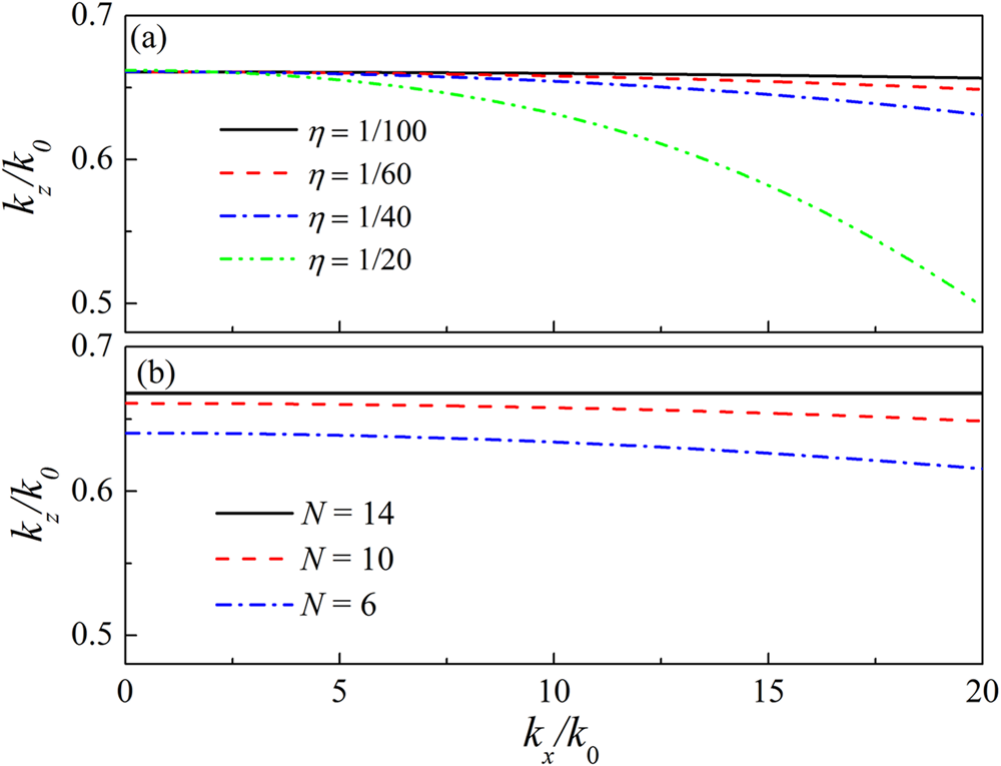
Equifrequency contour plot of the LMMD media for different values of (**a**) $\eta$ and (**b**) *N*.

Let us continue to discuss the NDP properties of the LMMD medium slab. It is well known that the NDP of the wave beams exhibits equifrequency contours (EFCs), and the long and flat EFCs correspond to a more efficient NDP. The layered LMMD medium is a periodic structure along the *z-*axis with the period $\Lambda =\eta {\lambda }_{0}$. According to Bloch’s theorem, the field amplitude matrix has ${\boldsymbol{\Psi }}(z+\Lambda )={e}^{i{k}_{z}\Lambda }{\boldsymbol{\Psi }}(z)$. Moreover, according to the transfer-matrix method, ${\boldsymbol{\Psi }}(z+\Lambda )=T{\boldsymbol{\Psi }}(z)$. Substituting ${\boldsymbol{\Psi }}(z+\Lambda )={\boldsymbol{T}}\Psi (z)$ into ${\boldsymbol{\Psi }}(z+\Lambda )={e}^{i{k}_{z}\Lambda }{\boldsymbol{\Psi }}(z)$, we obtain $|{\boldsymbol{T}}-{e}^{i{k}_{z}\Lambda }{\boldsymbol{I}}|=0$. Here, ***I*** is a $2\times 2$ unit matrix. Thus, the EFCs can be obtained by solving the eigenvalues of the matrix ${\boldsymbol{T}}$. Figure 4(a) shows the EFCs of the LMMD medium with ${\rho }_{m}=0.05+0.05i$ and $\delta \rho =0.025$, which are obtained by imposing the above Bloch condition on the field amplitudes evaluated through the TMM. The solid, dashed, dash-dotted, and dash-dot-dot lines represent the EFCs for $\eta =1/100$, $\eta =1/60$, $\eta =1/40$, and $\eta =1/20$, respectively. When $\eta =1/100$, the (${k}_{z}/{k}_{0}$) of the LMMD medium almost remains constant for the various (${k}_{x}/{k}_{0}$), indicating perfect NDP. It is obvious that the efficiency of the NDP in the LMMD medium will be reduced with increasing $\eta$. As $\eta$ increases to 1/20, the EFC of the LMMD medium effectively deviates from the constant non-diffracting value of (${k}_{z}/{k}_{0}$) approximately for $|{k}_{x}| > 5{k}_{0}$, and hence, the diffraction is not fully suppressed. Note that, according to the LMMD EMT, the ${k}_{z}$ $({k}_{z}={k}_{0}\sqrt{{\rho }_{r}^{eff}})$ of LMMD medium should remain a constant value for any ${k}_{x}$, thereby ensuring perfect NDP. Therefore, the proposed EMT and the NDP for the LMMD medium are not suitable for the case with a large $\eta$, especially for evanescent waves. We also examine the influence of the number of layers *N* in the unit cell on the NDP of the LMMD medium. In Fig. 4(b), the solid, dashed, and dash-dotted lines represent the EFCs of LMMD medium with the number of layers *N* of 14, 10, and 6, respectively. Here, ${\rho }_{m}=0.05+0.05i$, $\delta \rho =0.025$, and $\eta =1/60$. As *N* = 14, the EFC of the LMMD medium is very long and flat. It is found that the efficiency of NDP of the LMMD medium is reduced with decreasing number of layers, as shown in Fig. 4(b).

**Figure 5:**
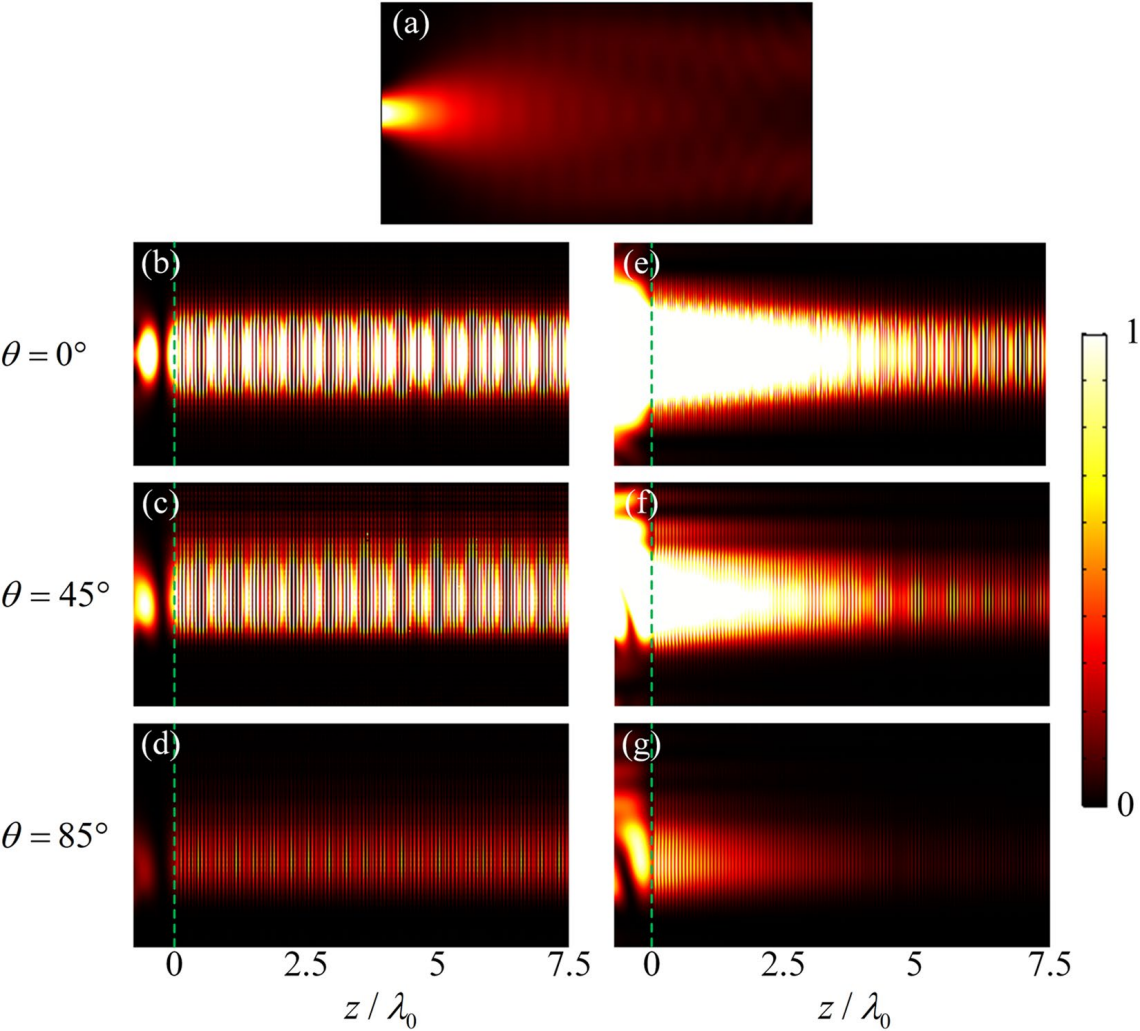
(**a**) Acoustic wave propagates in air. Acoustic waves propagate in the lossless LMMD media with incident angles of (**b**) 0°, (**c**) 45°, and (**d**) 85°. Acoustic waves propagate in the lossy LMMD media with incident angles of (**e**) 0°, (**f**) 45°, and (**g**) 85°. The dashed lines indicate the air-LMMD interfaces.

**Figure 6:**
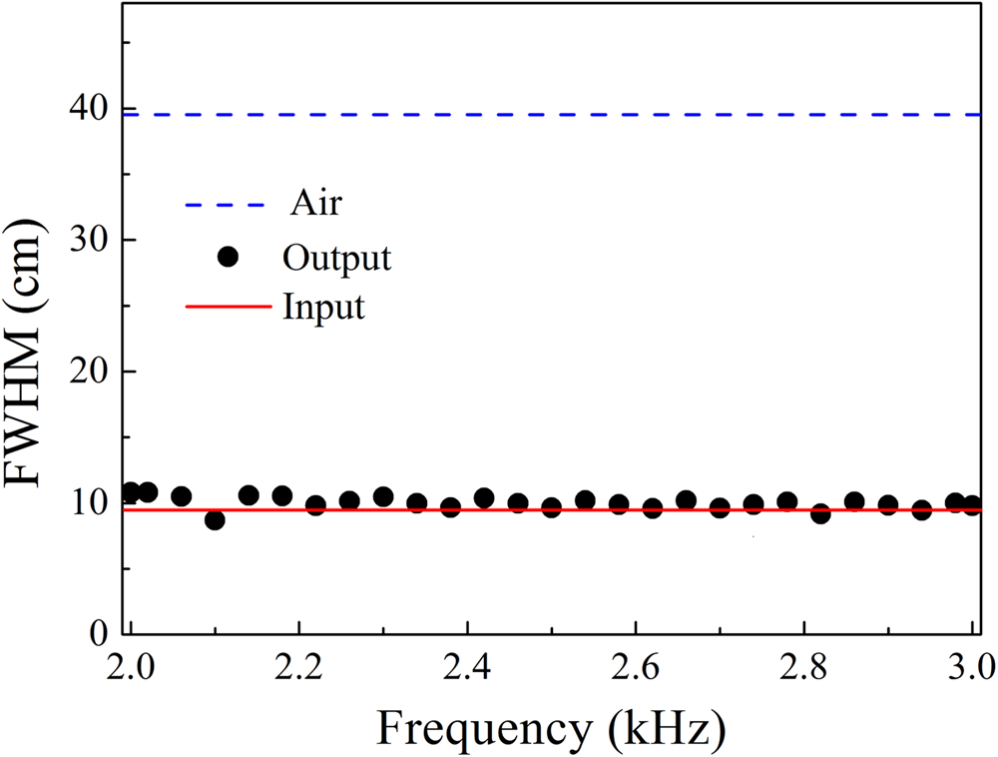
FWHMs of the input waves and FWHMs of the output waves through the LMMD medium as a function of frequency. The dashed line shows the FWHMs of the Gaussian waves after propagating a two-wavelength distance in air.

Finally, full-wave simulations of the finite element method (FEM) are used to simulate wave propagations in the LMMD medium. Figure 5(a) shows the acoustic wave propagation in air. The Gaussian beam propagates from the left side in the air, and the width of the wave beam broadens appreciably over the propagation distance. Figure 5(b),(c), and (d) show the Gaussian beam launched from a distance of 0.73 ${\lambda }_{0}$ into the LMMD medium without absorption at the incident angles of $0^\circ$, $45^\circ$, and $85^\circ$, respectively. Here, ${\rho }_{m}=0.05$ and *δρ* = 0. Figure 5(e),(f), and (g) represent the Gaussian beam propagating from air into the LMMD medium with absorption at the incident angles of 0°, 45°, and 85°, respectively. Here, ${\rho }_{m}=0.05+0.05i$ and *δρ* = 0.025. The source is aimed at the centre of the left air-LMMD interface of the structure when the incident angle is 0° and at the bottom corner when the incident angle is 45° or 85°. It can be seen clearly that the Gaussian beam propagates through the LMMD medium without a visible divergence, even when the incident angle is as large as 85°, and the diffractions are fully suppressed both in the lossless and lossy LMMD media. Therefore, all-angle NPD can be realized in the LMMD medium, which is also robust against the medium losses. In addition, we investigate the frequency response of the LMMD medium. Figure 6 shows the full widths at half maximum (FWHMs) of the input Gaussian waves (solid line) and the output waves (scattered circles) through the LMMD medium (thickness of 7.5 ${\lambda }_{0}$) as a function of frequency. By contrast, the dashed line represents the FWHMs of Gaussian waves after propagating a two-wavelength distance in air. The Gaussian waves with frequencies from 2 kHz to 3 kHz will be tested. The structural parameters of the layered LMMD medium used are the same as those for 2.5 kHz, which is chosen only for convenience. It is found that the FWHMs of the output waves through the LMMD medium are almost unchanged compared to those of the input waves, whereas the FWHMs of Gaussian waves will reach 40 cm after propagating only a two-wavelength distance in air. Therefore, the NDP in the LMMD medium could be well maintained in a broadband range.

## Discussion

The proposed LMMD EMT based on the first-principles method can well explain the broad-band all-angle acoustic non-diffraction propagation in the LMMD medium. The effective mass density of the proposed LMMD EMT is the sum of the average mass density and a contribution from the rapidly varying part of the density modulation. The rapid and large modulation of mass density should strongly suppress the transverse component of the velocity field and slow down diffraction of acoustic waves. As the modulation of the mass density becomes large in comparison with the mean value, the acoustic wave evolution will be affected not only by the value of the mass density but also by additional important contributions arising from the rapidly varying periodic density oscillations. Under this condition, the standard EMT is unsuitable for the layered LMMD medium, even within the longwave approximation regime.

The LMMD medium based on our theory is composed of layered fluid-like metamaterials with different mass densities. Fluid-like metamaterials can dynamically behave (in the homogenization limit) as true fluid materials. Many methods have been proposed to realize fluid-like metamaterials. For example, Torrent and Sánchez-Dehesa^26^ proposed a design of a fluid-like metamaterial based on the homogenization properties of a solid structure composed of cylindrical scatters. Li *et al*.^31^ experimentally used brass fins embedded on a brass substrate to realize a fluid-like metamaterial. A 2D multilayered fluid-fluid structure can be obtained inside a planar wave guide made of aluminium, in which a circular cavity is drilled with an embedded corrugated structure^32^. Moreover, fluid-like metamaterials with negative mass density can be obtained by using membrane-type metamaterials with simple constructs^33, 34^. Furthermore, the recent rapid increases in additive manufacturing and nanoscale manufacturing are very beneficial to fabricating complex and small-scale metamaterials^25^. Therefore, we believe that the multilayered LMMD medium could be realized by fluid-like metamaterials.

In conclusion, an LMMD medium has been proposed that is capable of realizing the non-diffraction propagation of acoustic waves. An EMT for the LMMD medium was derived from the first-principles method via the transfer-matrix method for both propagating and evanescent waves. It is found that the NDP in the LMMD medium is independent of the incident angle and can be operated in a broad-band manner. The influence of the geometry on the NDP in the LMMD media was investigated in detail. A smaller *η* and a larger *N* can ensure longer and flatter EFCs of the LMMD medium, indicating better NDP. This stratified medium may be useful for subwavelength imaging, wave beaming, and acoustic waveguides.

## Methods

The numerical simulations are performed by using the finite element method based on COMSOL Multiphysics software. The background medium is air, which has mass density and speed of sound of 1.25 kg/m^3^ and 343 m/s, respectively. The frequency of the incident acoustic waves is 2.5 kHz in the finite element calculations. An LMMD medium slab with $L=450\eta {\lambda }_{0}$ is investigated, and the parameters are *N* = 10 and $\eta =1/60$. The unit cell with a thickness of $\Lambda =\eta {\lambda }_{0}$ consists of *N* homogeneous layers. The mass density of the *j*th layer in the unit cell ($j=1,\ldots ,10$) along the *z* direction is given according to Eq. (10). The Gaussian beam with a FWHM of 9.6 cm propagates from the air into the LMMD medium. The average of the relative mass density *ρ*_*r*_ is ${\rho }_{m}$, where ${\rho }_{m}=0.05$ and $\delta \rho =0$ for the lossless case and ${\rho }_{m}=0.05+0.05i$ and $\delta \rho =0.025$ for the lossy case in the numerical simulations. Periodic boundary conditions are imposed in the *x* direction, and radiation boundary conditions are set for the remaining boundaries. The largest mesh element size is lower than one tenth of the incident wavelength, and the further refined meshes are applied in the domain of the unit cells of the microstructure.
